# Implementation of a data use strategy in situation rooms in two metropolitan areas of Honduras in the context of COVID-19

**DOI:** 10.1093/oodh/oqae007

**Published:** 2024-05-06

**Authors:** Freddy Hidalgo, Liziem Valladares, Diana Gonzalez, Lorenzo Pavón

**Affiliations:** Data.FI, Palladium, Torre Alfa 2° nivel, Tegucigalpa, Honduras 11101; Data.FI, Palladium, Torre Alfa 2° nivel, Tegucigalpa, Honduras 11101; Data.FI, Palladium, 1331 Pennsylvania Avenue, NW, Suite 600, Washington, DC 20004, USA; Health Surveillance Unit, Ministry of Health, 4Q4R+8R3, Avenida Miguel Cervantes, Tegucigalpa, Francisco Morazán, Honduras

**Keywords:** data use, decision making, accountability, digital health enabling environment, digital health, epidemic control rooms

## Abstract

The U.S. Agency for International Development’s Data for Implementation project supported the Ministry of Health in Honduras’ Central District and San Pedro Sula health regions to implement a new situation room methodology to analyze data and make timely, informed decisions during the pandemic. This mixed-methods study explored if the new methodology contributed to increased workforce capacity in data use and improved leadership and governance at the regional level from 2022 to 2023. We administered a self-assessment questionnaire and semi-structured interviews to situation room participants, regional health authorities and Data.FI staff to unveil perceptions around mastery, proficiency and confidence of data use knowledge and skills and to explore any perceived changes in the digital health enabling environment. After one year of situation room implementation, most participants reported being ‘competent’ or ‘expert’ in analysis and data visualization (59%) and being ‘very confident’ or ‘confident’ in applying data use competencies (83–93%). The thematic analysis revealed five themes indicating changes in the enabling environment. Three of the themes—data analysis and visualization, peer collaboration and replicability—fall under the World Health Organization’s workforce eHealth building block; two of the themes—leadership and governance of health data—fall under the leadership and governance eHealth building block. This study demonstrates the value of situation rooms as a capacity strengthening intervention and as an opportunity for use during new health emergencies.

Abrégé

Le projet « Data for Implementation » de l’Agence des États-Unis pour le développement international (USAID) a permis au département de la santé des régions sanitaires du district central et de San Pedro Sula du Honduras de mettre en œuvre une nouvelle méthodologie des centres opérationnels stratégiques pour analyser les données et prendre des décisions éclairées et opportunes pendant la pandémie. Cette étude à méthodes mixtes visait à déterminer si la nouvelle méthodologie avait contribué à accroître les capacités des effectifs en matière d’utilisation des données et à améliorer le leadership et la gouvernance à l’échelle régionale de 2022 à 2023. Nous avons soumis aux participants des centres opérationnels stratégiques, aux autorités sanitaires régionales et au personnel de Data.FI un questionnaire d’autoévaluation et nous avons mené des entretiens semi-structurés auprès d’eux pour cerner leurs perceptions concernant la maîtrise, l’aptitude et la confiance des connaissances et des compétences en matière d’utilisation des données et pour étudier tout changement perçu dans l’environnement propice à la santé numérique. Après un an de mise en œuvre en centre opérationnel stratégique, la plupart des participants se sont déclarés « compétents » ou « experts » en analyse et visualisation de données (59%) et « très confiants » ou « confiants » dans l’application de leurs compétences en matière d’utilisation des données (83 à 93%). L’analyse thématique a révélécinq thèmes indiquant des changements dans l’environnement favorable. Trois de ces thèmes (analyse et visualisation des données, collaboration entre pairs et reproductibilité) relèvent des piliers de la santé numérique des effectifs de l’Organisation mondiale de la santé (OMS); deux des thèmes (leadership et gouvernance des données de santé) relèvent du module leadership et gouvernance de la santé en ligne. Cette étude démontre la valeur des centres opérationnels stratégiques en tant qu’interventions de renforcement des capacités et en tant qu’occasions d’utilisation lors de nouvelles urgences sanitaires.

Resumen

El proyecto Data for Implementation de la Agencia de los Estados Unidos para el Desarrollo Internacional (USAID) asistió a la Secretaría de Salud de Honduras en las regiones sanitarias del Distrito Central y San Pedro Sula para implementar una nueva metodología de salas situacionales con el fin de analizar datos y adoptar decisiones oportunas e informadas durante la pandemia. En este estudio de métodos mixtos se examinó si la nueva metodología contribuyó a aumentar la capacidad del personal en el uso de datos y a mejorar el liderazgo y la gobernanza a nivel regional de 2022 a 2023. Administramos un cuestionario de autoevaluación y entrevistas semiestructuradas a los participantes de las salas situacionales, las autoridades sanitarias regionales y el personal de Data.FI para descubrir cuáles eran las percepciones sobre el dominio, la competencia y la confianza en el uso de datos, los conocimientos y las habilidades, y para analizar los cambios percibidos en el entorno propicio para la salud digital. Después de un año de implementación de las salas situacionales, la mayoría de los participantes declararon ser ‘competentes’ o ‘expertos’ en análisis y visualización de datos (59%) y sentirse ‘muy seguros’ o ‘seguros’ en la aplicación de las competencias de uso de datos (83–93%). El análisis temático reveló cinco temas que indican cambios en el entorno propicio. Tres de los temas (análisis y visualización de datos, colaboración entre pares y replicabilidad) corresponden al componente de recursos humanos de los sistemas de salud según la Organización Mundial de la Salud; dos de los temas (liderazgo y gobernanza de los datos de salud) corresponden al componente de liderazgo y gobernanza. Este estudio demuestra el valor de las salas situacionales como intervención de fortalecimiento de la capacidad y como oportunidad para su uso durante nuevas emergencias sanitarias.

## INTRODUCTION

The COVID-19 pandemic revealed the need for more effective coordinating mechanisms to analyze and use available data to make timely, informed decisions for a health emergency. Situation rooms—also known as epidemic control rooms—were implemented by ministries of health globally to support improved public health management and evidence-based decision-making.

The concept of health-oriented situation rooms derives from the military or emergency response ‘war rooms’ where key high-level decision-makers from across government functions meet in one location and are provided with updated data and analyses that enable them to make time-sensitive, critical decisions jointly based on the best available information. In Latin America, the application of situation rooms to the health sector is not new; the first situation room in Latin America was put in place in Brazil in 1994 and other countries—like Nicaragua, Cuba and Peru—followed shortly thereafter. These were used to monitor health decentralization processes, surveillance indicators and emergencies [1]. Similarly, across sub-Saharan Africa, epidemic control rooms have been repurposed in recent years to encourage improved decision-making to address the HIV response through investments by the Joint United Nations Programme on HIV/AIDS (UNAIDS) and U.S. Agency for International Development (USAID) [2, 3]. In Honduras, situation rooms had been used sporadically to present epidemiological data, mostly reviewing prevalence, incidence and mortality.

During the pandemic, the government of Honduras and international donors invested in pilots of digital tools, including DHIS2 Tracker in the two largest metropolitan regions—the Central District and San Pedro Sula—to digitize client-level immunization data, which was previously recorded on paper. Despite these efforts, the SIVAC computerized vaccination system, which reported aggregated data, and paper-based forms prevailed. As an alternative solution, USAID, through the Data for Implementation (Data.FI) project, supported the Ministry of Health (MOH) to strengthen data use and coordination for the COVID-19 response leveraging the available data for decision-making. This involved agreement on a common logic framework and the adoption and implementation of a National Data Use Strategy in an existing subnational situation room in San Pedro Sula and the establishment of a new room in Tegucigalpa [4].

Before Data.FI’s intervention in early 2022, situation room meetings were exclusively led by the MOH’s Health Surveillance Unit, lacked a defined methodology and rarely led to actions or commitments. The situation room in San Pedro Sula received funding during the pandemic from the Pan American Health Organization to buy furniture and technological equipment to facilitate the data review meetings; however, the situation room was not operational prior to the Data.FI intervention. With Data.FI’s support, the situation rooms were developed to enable structured and sustained review of regional COVID-19 data from digital systems and continual problem-solving on COVID-19 priorities, focusing mostly on COVID-19 vaccination since mid-2022.

Through stakeholder workshops (following a new training curriculum developed by Data.FI) and ‘side-by-side’ capacity strengthening, Data.FI staff worked closely with national and regional health authorities to develop and implement a new situation room methodology. The approach encouraged cross-sectoral collaboration and data use leading to actions to interrupt COVID-19 transmission and expand vaccination coverage. The new situation room methodology encompassed five key elements: (1) multidisciplinary problem solving, (2) concise scheduling―with short, frequent meetings, ideally weekly 1-hour meetings, (3) a focus on data visualization, (4) use of quality improvement (QI) methods and (5) action planning for accountability. Data are presented in several forms in situation rooms—tables, graphs, maps, technical documents and strategic reports—to discuss and guide decisions and planning. With the new methodology, data are shared, validated and analyzed quickly using digital systems and MOH subnational staff are supported to plan, program, and monitor activities, prioritize problems and recommend ways to address them. After implementation of the two regional situation rooms, a national situation room initiative emerged in September 2022 under the leadership of the Health Surveillance Unit of the MOH with support from Data.FI.

**Figure 1:**
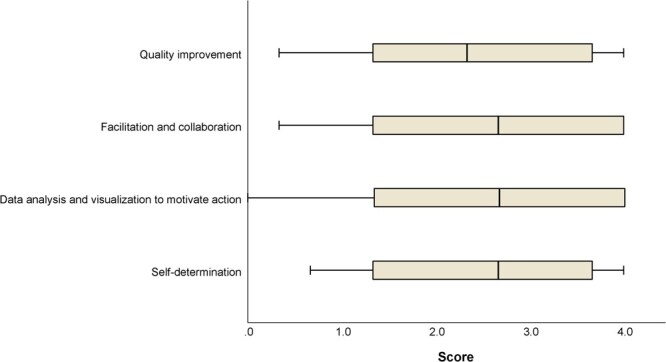
Mastery of data use knowledge and skills (median and inter-quartile range) (N = 29) Note: Fig. 1 illustrates the distribution of the total scores corresponding to the four questions within each of the four areas. Each question’s score was measured on a scale from 0 to 1. For instance, if a participant reported to have been trained, observed, and practiced (0.33 + 0.33 + 0.33 = 1) to the four questions in the area of quality improvement, then the participant would have a score of 4.

As part of the USAID COVID-19 Vaccine Digital Health Collaborative Learning Agenda, USAID and implementing partners co-developed a theory of change aiming to capture the different pathways in which digital and data system interventions implemented during the pandemic could have led to COVID-19-specific outcomes and impacts, as well as outcomes and impacts on the digital health enabling environment and health system [5, Figure 1]. Framed within this theory of change, this study aimed to understand how and to what extent the implementation of situation rooms―as data use structures―had immediate outcomes in terms of data quality, analysis and use. It also explored if there were indications of a strengthened digital health enabling environment in addition to the COVID-19-specific outcomes and impacts documented elsewhere through data use cases [6–8]. Although data use is not called out separately as a World Health Organization (WHO) eHealth building block [9], Werner et al. advocate for the promotion of a culture of data use and feedback on data and data use to be considered as key elements of the digitalization process [10].

When the Data.FI project launched, it defined data use as the process of data review whereby decision-makers explicitly consider routine aggregate, client-level and/or survey data, take an action and are able to witness a change in performance as a result [11]. This study will contribute to elucidating why workforce capacity strengthening for using quality evidenced-based data for decision-making is vital for a digital health enabling environment. As the Data Demand and Information Use framework proposed by the MEASURE Evaluation project suggests, ‘The more positive experiences a decision maker has using information to support a decision, the stronger will be the commitment to improving the timeliness of data collection systems’ [12]. We hypothesized that once decision-makers distill the value of quality routine data for making policy and programmatic decisions, and staff skills in data quality, analysis and use improve, their attitudes and practices will change by stimulating greater demand for data and decreasing their resistance to adopt new digital technologies. As such, workforce capacity strengthening in data use might become a pre-condition for the successful implementation of national digital health strategies and thus for overall health system strengthening. Without a data use culture, the transition to new digital tools, systems and platforms will mostly be seen as overload for the workforce and mainly donor-driven due to the lack of domestic funds to implement the national digital agenda [13].

The objective of this study was to measure and analyze the extent to which Data.FI support contributed to a strengthened digital health enabling environment through improved regional leadership, governance of health data and workforce data use capacity after the implementation of COVID-19 situation rooms in the Central District and San Pedro Sula metropolitan health regions in Honduras during the 2022 to 2023 period. Specifically, we examined:

1. To what extent have situation room participants leveraged Data.FI data use capacity strengthening?
2. How do participants perceive their own proficiency and confidence to apply data use competencies?
3. What improvements have been made in workforce data use capacity at the organizational level to plan and make evidence-based decisions in the context of COVID-19?
4. Have Data.FI interventions resulted in improvements in leadership and governance?
5. What is the capacity of situation room staff to replicate the new situation room methodology to respond to other public health events or future threats?

## METHODS

### Study design

This was a mixed-methods study consisting of a guided written self-assessment and semi-structured interviews. The study was conducted in the two main health regions of Honduras: the Central District, which includes the country's capital in Tegucigalpa, and San Pedro Sula, the industrial capital.

We developed and used a paper-based structured questionnaire for respondents to report their mastery of data use knowledge and skills and their self-assessed proficiency and confidence levels in applying their data use knowledge and skills. This data collection tool was designed by the study team probing aspects of Data.FI's data use training curricula, which consisted of eight elements: (1) action planning and prioritization, (2) facilitation for consensus, (3) creating meaningful visuals, (4) QI methods (for example, the Plan-Do-Study-Act [PDSA] cycle and root cause analysis), (5) comparing performance with targets, (6) identification of performance issues, (7) critical thinking and (8) questioning.

The questionnaire consisted of 50 questions in total. The first section of the self-assessment tool asked for participants' basic sociodemographic information (five closed-ended questions). The second section of the questionnaire (16 multiple-choice questions) examined mastery of data use knowledge and skills, where respondents were asked to mark if they had been trained, have observed, and have practiced data use competencies in the four main areas of analysis: (1) self-determination (ownership, commitment, leadership, and motivation), (2) data analysis and visualization to motivate action, (3) meeting facilitation and collaboration and (4) quality improvement. These four areas were selected and adapted after a review of existing capacity building frameworks (see Table 1 for definitions and mapping to training areas) [15–17]. In this section, for each question participants had to mark their perceived level of proficiency: beginner, advanced beginner, competent or expert or mentor.

**Table 1:** Four self-assessment analysis areas

Analysis area	Definition	Data use training curriculum areas
1. Self-determination	To promote ownership, commitment, confidence, motivation, leadership, and self-direction that catalyzes the other capacities [14]. This definition includes:• Data-informed leadership and advocacy• Strategy development and planning• Priority-setting and decision-making• Accountability• Motivation and self-direction• Dissemination	Action planning and prioritization
2. Data analysis and visualization to motivate action	To carry out technical tasks related to data analysis, questioning (defined in this study as the ability to pose a series of questions to explore data meaning and its implications in policy and implementation), visualization, interpretation, and synthesis	Creating meaningful visuals Identification of performance issues
3. Meeting facilitation and collaboration	To facilitate meetings to reach consensus and incentivize stakeholders to collaborate, network, and communicate with national and local levels	Facilitation for consensus Questioning
4. Quality improvement (QI)	To know and apply QI concepts and methods for data analysis and strategy planning	QI methods Critical thinking Comparing performance with targets

The third section of the tool asked participants to rate their confidence in applying their data use knowledge and skills (25 Likert scale questions), adapting seven data analysis and use indicators from USAID’s Health Policy Project’s Organizational Capacity Assessment Suite of Tools [18]. The fourth section asked for additional information (four open-ended questions).

Qualitative data was collected using three semi-structured interview guides, one for situation room participants (eight open-ended questions), one for regional authorities (14 open-ended questions) and one for Data.FI staff (12 open-ended questions). The semi-structured qualitative interview guides sought to capture respondents’ perspectives on organizational changes in the health regions that resulted from the Data.FI situation room interventions in terms of: (1) workforce capacity strengthening in data use, (2) effective stakeholder engagement and coordination, and (3) quality improvement. Interviews also probed the eight data use capacities that Data.FI provided training on during its intervention and asked about leadership and governance of health data to understand if there were indications of sustained changes in the enabling environment after implementing the new methodology. Interviews also probed whether, after Data.FI’s intervention, respondents would feel confident replicating the new situation room methodology in the context of other diseases or health threats.

### Sampling

The study team identified 16 situation room participants in the Central District and 20 in San Pedro Sula based on convenience sampling to complete the written self-assessment (36 participants identified in total). All target respondents were MOH staff and included both the regional director and the regional director of the Health Surveillance Unit, who also actively participated in the situation rooms.

Six situation room participants from each region were randomly sampled to be asked to participate in a semi-structured interview (we anticipated conducting 18 interviews in total). The universe of situation room participants included those whose frequency of participation since the implementation of Data.FI's situation room methodology was moderate (defined as between six and eight sessions) or high (defined as more than eight sessions). Individuals who at that moment were no longer active participants in the situation room sessions were also considered as long as they continued to work at the MOH regional office. If a selected participant refused to participate, we planned to randomly select another name from the list. We also planned interviews with the regional director and the regional director of the Health Surveillance Unit in each region and with the two Data.FI data use technical advisors who attended the situation room meetings since their launch in early 2022. Interviews were planned to be conducted in-person by Data.FI staff.

### Data collection

Data collection took place between May 15 and June 7, 2023. The principal field investigator applied both data collection tools in close coordination with the study team. The data collection tools were developed in English and then translated into and administered in Spanish. The principal field investigator, the director of the national Health Surveillance Unit (a coinvestigator in this study), and MOH staff from the Health Surveillance Unit piloted the data collection tools and administered the paper-based questionnaire in person in MOH facilities in both regions. Interviews took place either physically in MOH facilities (n = 10) or virtually via Microsoft Teams (n = 4). The principal field coinvestigator contacted interviewees by phone and explained the study objective using an information sheet; later, he conducted individualized semi-structured interviews following interview guides. The US-based principal investigator participated in three out of four virtual interviews including the two interviews with Data.FI staff.

The written self-assessment lasted on average 20 minutes, while interviews lasted 7 to 45 minutes and were audio recorded and transcribed verbatim by the research team. The seven-minute interview was interrupted by the seizure of health facilities in the Central District where the interview was taking place (during a period of political turmoil). As it was impossible to reschedule the interview, it was excluded from the analysis. Due to logistical challenges and availability of situation room participants, data collection was not conducted sequentially as originally planned, with self-assessment followed by interviews.

Participant recruitment for the facilitated self-assessment was by convenience. All situation room participants and the two regional health authorities were eligible to complete the self-assessment. However, given some political disturbances taking place during data collection, fewer persons than anticipated were available in the region to complete the self-assessment. Questionnaire data was captured in Excel and imported into SPSS for analysis.

We used a hybrid approach for recruitment of interview participants. Researchers selected the two main regional authorities (regional director and director of the regional Health Surveillance Unit) and six MOH staff for each region. The latter were initially randomly sampled, replacing dropouts with convenience sampling. One additional person was interviewed upon request.

All participants provided written and verbal informed consent to participate in the study and were informed that their responses might be published anonymously. Participants did not receive any benefit for participating in this study beyond any psychological benefits possibly associated with sharing their insights and experiences.

### Data analysis

#### Self-assessment survey

Quantitative data were analyzed using SPSS to produce descriptive statistics. To synthesize the analysis, we created three variables: mastery of data use knowledge and skills, self-assessed proficiency and self-assessed confidence levels in applying their data use knowledge and skills.

For each item related to mastery of data use knowledge and skills, the study team assigned equal weights corresponding to the level of mastery. For example, a respondent who indicated they were trained, observed, and practiced a specific skill was assigned a score of 1, whereas a respondent who indicated they were only trained was assigned a score of 0.33. The average score was then calculated for each of the four analysis areas described in Table 1. We defined proficiency as the level of expertise in data use self-reported by participants (beginner, advanced beginner, competent or expert or mentor) and confidence as the degree of certainty that participants reported putting into practice the new or refreshed data use competencies. Confidence acts as a proxy for replicability. Proficiency and confidence levels were reported as percentages. Average scores and summary statistics were obtained for each of the four analysis areas; individual question items were analyzed separately.

#### Semi-structured interviews

The qualitative data was analyzed in QSR NVivo 14 software using pre-defined and emergent themes. The study team met to collaboratively develop a codebook. The principal investigators read a small set of interview transcripts and coded the data, applying the codebook. We ensured intercoder reliability by discussing the coding of the first few transcripts, including inconsistencies, redundancies and code definitions. The codebook was then revised based on discussions. Two coders independently applied the codebook, one of them coded 10 interviews and the other four. Next, the team held meetings with a third coinvestigator to discuss and categorize the interview content. The study team translated the quotes back to English for inclusion in this paper. Table 2 summarizes the main definitions used to organize our thematic analysis.

**Table 2:** Main codebook pre-defined definitions

Theme	Definition
Leadership	The ability to: (1) direct and coordinate digital health, ensure alignment with health goals and political support, and promote awareness and engage stakeholders, (2) use mechanisms, expertise, coordination, and partnerships to develop or adopt eHealth components (e.g. standards), and (3) support and empower required change, implementation of recommendations, and monitoring of results for delivery of expected benefits [9].
Governance	The ability to respect hierarchical structures and comply with standardized processes, procedures, and practices for provision of health services [19].
Capacity	The knowledge, skills, and motivations, as well as the relationships that enable an actor—an individual, an organization, or a network—to take action to design and implement solutions to local development challenges, to learn and adapt from that action, and to innovate and transform over time [20].

**Table 3:** Self-assessment respondent characteristics

**Variable**		**Health region**		**Total** **N (%)**
		**San Pedro Sula** **N (%)**	**Central District** **N (%)**	
Sex	Male	1 (6.7)	6 (42.9)	7 (24.1)
	Female	14 (93.3)	8 (57.1)	22 (75.9)
Age	18–30 years	0 (0.0)	1 (7.1)	1 (3.4)
	30–40 years	10 (66.7)	7 (50.0)	17 (58.6)
	40–50 years	4 (26.6)	4 (28.6)	8 (27.6)
	More than 50 years	1 (6.7)	2 (14.3)	3 (10.3)
Maximum degree of studies	Bachelor’s degree or technical school	9 (60.0)	4 (28.6)	13 (44.8)
	Master’s degree	6 (40.0)	10 (71.4)	16 (55.2)
Duration of participation in the situation rooms	Less than 3 months	0 (0.0)	3 (21.4)	3 (10.3)
	Between 3 and 6 months	0 (0.0)	2 (14.3)	2 (6.9)
	Between 6 and 9 months	5 (33.3)	2 (14.3)	7 (24.1)
	More than 9 months	10 (66.7)	7 (50.0)	17 (58.6)
Role in situation room	Regional health authority	1 (6.7)	1 (7.1)	2 (6.9)
	Situation room participant	14 (93.3)	13 (92.9)	27 (93.1)

### Ethics

Ethical review for this study was conducted by the Ethical Committee of the Master’s in Public Health Association of Honduras. All study protocols and materials were reviewed and approved by the Health Surveillance Unit of the Honduran MOH and USAID.

The informed consent process for interview participants was individualized and private; the field-based principal investigator privately shared information about the study with each potential participant and sought documented informed consent. There was little to no risk to participants involved in this study. For Data.FI staff, the study team ensured that all questions were asked in a nonjudgmental and unbiased manner. Participants were informed that they could stop participation at any point or choose not to answer specific questions.

## RESULTS

### Participant characteristics

#### Self-assessment

Twenty-nine persons completed the self-assessment questionnaire: 15 in San Pedro Sula and 14 in the Central District. Overall, about 76% of participants were female (22), and 59% were in the 30- to 40-year age range (17). All had either a master's degree (55%) or bachelor's degree (45%). More than 80% of respondents had participated in the situation room for more than six months at the time of the interview (59% for more than nine months and 24% for between 6 and 9 months). Compared to the Central District, participants in San Pedro Sula were mostly female (93% vs 57%), younger (67% under 40 years of age vs 57%), had a lower maximum degree of education (40% with a master’s degree vs 71%) and had participated for a longer period in the situation room (100% participating for 6 months or longer vs 64%). Respondents were primarily technical staff (93%), also referred to as situation room participants; the other 7% were regional authorities (only one of the two regional authorities in each region completed the self-assessment). Table 3 summarizes the characteristics of the self-assessment respondents.

#### Semi-structured interviews

Interviews were conducted with 14 participants: four regional authorities (29%), eight technical staff (57%), and two Data.FI data use technical advisors (14%). Eight interviewees (57%) represented San Pedro Sula health region and six (43%) represented the Central District; 11 (79%) were female; 10 (71%) had a master’s degree; and all (100%) participated in the situation room for more than 6 months. Nine of the fourteen interviewees had previously completed the self-assessment questionnaire. Table 4 lists interviewee characteristics.

**Table 4:** Semi-structured interview respondent characteristics

**Variable**		**Health region**		**Total** **N (%)**
		**San Pedro Sula** **N (%)**	**Central District** **N (%)**	
Sex	Male	1 (12.5)	2 (33.3)	3 (21.4)
	Female	7 (87.5)	4 (66.7)	11 (78.6)
Age	30–40 years	5 (62.5)	2 (33.3)	7 (50.0)
	40–50 years	1 (12.5)	2 (33.3)	3 (21.4)
	50–60 years	0 (0.0)	1 (16.7)	1 (7.1)
	More than 60 years	2 (25.0)	1 (16.7)	3 (21.4)
Maximum degree of studies	Bachelor’s degree or technical school	4 (50.0)	0 (0.0)	4 (28.6)
	Master’s degree	4 (50.0)	6 (100)	10 (71.4)
Duration of participation in the situation rooms	Between 6 and 9 months	4 (50.0)	1 (16.7)	5 (35.7)
	More than 9 months	4 (50.0)	5 (83.3)	9 (64.3)
Role in situation room	Regional health authority	2 (25.0)	2 (33.3)	4 (28.6)
	Situation room participant	5 (62.5)	3 (50.0)	8 (57.1)
	Data.FI staff	1 (12.5)	1 (16.7)	2 (14.3)

### To what extent have situation room participants leveraged Data.FI data use capacity strengthening?

To answer the study's first question, we look at self-reported mastery of data use knowledge and skills. Participants reported median mastery of data use knowledge and skills scores between 2 and 3, on a scale of 1 to 4, in each of the four areas of analysis: self-determination, data analysis and visualization to motivate action, facilitation and collaboration and quality improvement (Fig. 1). Facilitation and collaboration, as well as data analysis and visualization, were the two key areas where respondents identified greater self-reported mastery. The other two areas, quality improvement and self-determination, showed homogenous behavior.

When analyzing the questions individually, the highest average scores corresponded to competencies for identifying probing questions (69%), defining significant data use (67%), designing visualizations that highlight performance issues (67%), identifying strategies to ensure stakeholder participation (67%) and communicating data use experiences to colleagues and authorities (67%). Findings showed that using the PDSA cycle to assess implemented actions in relation to expected results and adjusting them had the lowest average score (49%).

### How do participants perceive their own proficiency and confidence to apply data use competencies?

In regard to the second question, participants answered questions regarding their self-reported proficiency and confidence levels in the four analysis areas: self-determination, data analysis and visualization, facilitation and collaboration and quality improvement.

#### Self-reported proficiency to apply data use skills

**Figure 2:**
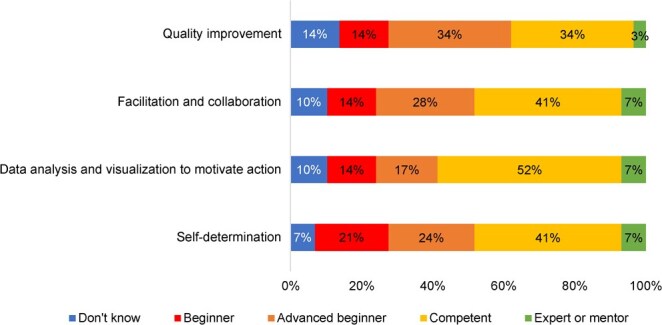
Self-reported proficiency level (N = 29)

Findings indicated that although the distribution by each analysis area for proficiency was similar, participants reported to be the most competent (52%) or expert (7%) in data analysis and visualization, followed by facilitation and collaboration and self-determination (each with 41% competent and 7% expert) (Fig. 2).

Three persons answered ‘don’t know’ or ‘don’t want to respond’ to most of the proficiency questions (all from the Central District and having participated in the situation room for different lengths of time; two had a master's degree). Another three participants, from both regions, rated themselves beginners across the four analysis areas. These participants were male, had a bachelor’s degree and had participated for over 6 months in the situation room. With a few exceptions, the three ‘beginner’ participants responded ‘observed’ to most of the mastery of data use questions, without any reported training and practice. Beginners clustered around the following topics: (1) using the PDSA cycle to assess implemented actions in relation to expected results and adjust them (31% of respondents, n = 9), (2) defining significant data use (20%, n = 6), (3) generating change ideas and corresponding action items (17%, n = 5) and (4) designing visualizations that highlight performance issues (17%, n = 5).

#### Self-reported confidence to apply data use skills

**Figure 3:**
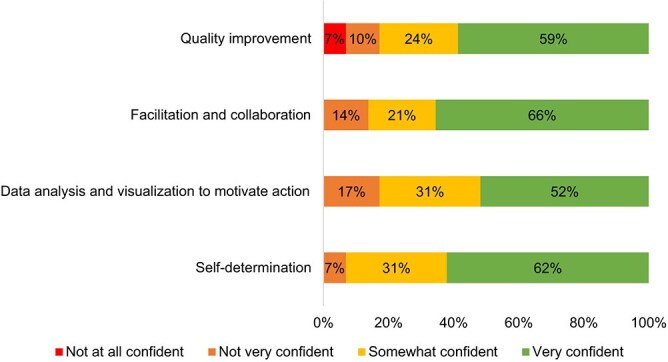
Self-reported confidence level (N = 29)

Self-reported confidence scores indicated that overall, participants felt somewhat or very confident using their data use knowledge and skills across each of the four analysis areas (between 83% and 93% of study participants) (Fig. 3). When analyzing individual questionnaire items, the following items had the largest percentage of participants who reported being very confident:

• Explain why priority setting is important for decision-making (75.9%)

• Communicate the findings of regional analyses to local authorities (e.g. to Network Coordinating Teams [ECORES] or health facilities) (75.9%)

• Participate in the creation of a situation room to respond to a new pandemic or health threat (75.9%)

In contrast, the following items had the largest percentage of participants who reported being not very confident, not at all confident or do not know/do not want to answer:

• Anticipating the next decisions and questions of health units, program managers, and planners about policies and programs that can be informed by data (20.7%)

• Using the action planning tool (accountability tool) to follow commitment and progress (20.7%)

• Applying the PDSA cycle in their own work (24.1%)

• Compiling information from different sources for analysis (31%)

• Doing a root cause analysis using the Ishikawa diagram (fish diagram) (31%)

#### Contributing interventions

The questionnaire also asked respondents to report which interventions contributed to their data use proficiency and confidence levels—52% of the 29 respondents attributed their level to the Data.FI situation room intervention. The remaining 48% referred to other government capacity strengthening activities led by the immunization program, the Health Surveillance Unit, or the quality unit of the MOH, as well as graduate studies paid for with their own resources.

### What improvements have been made in workforce data use capacity at the organizational level to plan and make evidence-based decisions in the context of COVID-19?

Semi-structured interviews provided insights into our third study question. Most MOH staff interviewed perceived the situation room as a new opportunity to receive training or refresh knowledge. One participant described how the situation room impacted his data use capacity in different aspects, including his soft skills: ‘I feel that situation rooms contributed in a positive way. It affected in different ways how I carried out my work, both in terms of data quality management, in the presentation of data, or being able to stand in front of a group of people and speak comfortably and eloquently.’ Some interviewees noted how soft skills and, in particular, public speaking was reinforced by observation: ‘Before I couldn't even speak in public, now I am like the Data.FI data use advisor.’

Overall, participants described positive changes across the data use curriculum training areas described previously (see Methods, Study design). Improvements highlighted by participants clustered around the themes of data analysis and visualization as well as peer collaboration, which are presented next.

#### Data analysis and visualization

Throughout the interviews, data analysis and visualization emerged as a key area of positive change, although some recognized that, at the same time, there was still room for improvement. Situation rooms were described by most as a turning point for the way the region analyzed their data and implemented action items. For instance, the region now analyzes not only regional-level data but also data from health networks and health facilities. Clear improvement in data analysis was illustrated by one respondent: ‘When we started, it took us 6 hours to analyze data to prepare a situation room, now we do it in less than 2 hours.’

Participants noted that using data analysis helped identify opportunities to optimize resources. Data analysis helped health regions identify and prioritize at-risk populations needing COVID-19 vaccination: ‘We said we have to find strategies to target elderly unvaccinated adults because we have analyzed the data in the situation room and know that they are the ones at risk.’ Another participant explained: ‘We observed in the analysis that it was likely for new waves to occur, so the region decided to keep some triages open … that was a useful strategy that eased the pressure on hospitals.’

Data analysis and visualization also helped participants perform data quality checks for completeness, accuracy, and consistency in reporting. Although one participant highlighted challenges in performing analysis with data provided by the Honduran Social Security Institute, in general, this is an area where participants reported that it supported their day-to-day work: ‘It has served me as a data quality control. Creating graphs helps us validate the data that we have officially reported.’ Another participant explained: ‘We thought we had a lower vaccination rate, and that was leading us to different decisions … having data, accurate data, helped us to focus on the issues that truly needed our attention.’ Other participant said, ‘Data.FI generated a positive conditioning, we can say that it forced individuals to make decisions based on real data, quality data.’ A Data.FI advisor stated, ‘One of the activities that contributed the most to capacity strengthening was … working with MOH staff on data analysis and performing data quality analysis with them.’

Interviewees also explained how the Data.FI intervention helped with presenting analyses in simple ways and applying standard visualization criteria. One participant said, ‘Before the situation rooms, the analysis used to be presented in paragraphs. Later we began to use tables, graphs and other visualizations that help us understand the issue at first sight. Someone who just joined the situation could easily visualize and interpret a graph… it helped us a lot.’ Another participant noted: ‘Data.FI proposed criteria unification, colors, font type, and other elements of visualizations; we were able to reach an agreement in which all the units work in a similar way with the same style… [this] helps us have a better understanding [among us].’

Accountability, defined as the ability of situation room participants to report progress to their peers and comply with duties assigned by regional leaders, was another area supported by data analysis and visualization. One Data.FI staff member interviewed said, ‘The most valuable aspect of the situation room intervention … the authorities looked at accountability with fresh eyes. The graph of interventions planned/in process/implemented was seen as an added value.’ A Data.FI staff member also described that the new methodology has helped MOH staff comply with weekly commitments as milestones toward their objectives: ‘Before, it [accountability and progress reporting] was seen as intrusive supervision, but now situation rooms openly speak about what works and doesn’t and are open to receive feedback and change.’ In addition, participants noted that accountability tracking promoted adaptive management, allowing the health regions to identify challenges and decide when to adapt and adjust strategies in a timely manner. Nevertheless, as one Data.FI staff member remarked, ‘The only thing that regional staff have not yet assumed is the task of holding the team accountable.’ Participants noted that in this regard, the role of an external party is valuable.

#### Peer collaboration

Improved teamwork or peer collaboration was an emerging theme; a perceived change in team integration and teamwork came across in several interviews. Participants acknowledged that, previously, information was not shared beyond their department (e.g. laboratory or surveillance). However, now that they clearly understand each person’s role and where each unit or department can help or collaborate, it is easier to jointly design strategies and plans or triangulate information to improve data quality and homologate targets. In particular, engagement between health networks and the surveillance unit was strengthened. A health authority explained how teamwork evolved:

‘At the beginning, health network staff were more withdrawn, but gradually became aware that this work had to be integrated with health surveillance and other units, social communication, journalists in institutional communications, well, everyone, the whole team… the administrator must be aware of why we need transportation, stationery, and equipment support. Legal services helped us understand how far we could go to search for information and the normative framework unit [helped us] understand roles and responsibilities.’

### Have Data.FI interventions resulted in improvements in leadership and governance?

Results related to the fourth study question, discussed below, also come from interviewees.

#### Leadership

In this study, we used the leadership definition included in the WHO’s National eHealth Strategy Toolkit (see Table 2). When asked about the benefits of the situation room approach related to leadership, interviewees highlighted that leaders empowered their staff to implement and sustain the intervention and noted that leaders worked beyond their regions to strengthen multilevel coordination and engage external stakeholders.

All Central District leaders, the regional director and the director of the regional Health Surveillance Unit described scheduling their regional weekly meetings just after the situation room to discuss situation room agreements with the wider regional team to assign responsibilities. One of the regional leaders in the Central District mentioned: ‘On Tuesdays, first we had the situation room meeting, then we held a team meeting. I tried to empower all the directors of units and departments to ensure continuity [of the situation room intervention]. We also involved the administrative department in addressing emerging needs, for example, when we needed transportation to retrieve information from a different location.’ Leaders said that having weekly team meetings after the situation rooms allowed them to cascade data analysis results to other technical staff and keep them aware of the regional health situation.

Leaders relied on situation rooms to train and empower MOH technical teams to sustain regional technical capacity*.* In addition to refreshing concepts and skills for the most seasoned staff, respondents said that situation rooms provided an opportunity for leaders to onboard new staff. Situation rooms allowed new MOH staff to learn how to analyze regional priorities in a collaborative way by observing how units and departments interacted with each other: ‘It is very important to mention that through situation rooms new staff have learned the importance of analyzing information, the relevance of using accurate data and how to use data in an adequate way to solve the problems that exist at the facility or the community level.’

Some interviewees noted leadership as the ability to convene other external actors, bringing them into the discussion, and the ability to promote coordination with nongovernmental organizations, donors, other public and private actors within the Honduras health system, private laboratories and others. One interviewee said, ‘Many times, we feel frustrated because we don’t have the inputs or the resources to implement strategies, but in the situation room, we can identify which actors outside the Ministry of Health or outside the region are doing the same thing that we are doing, and then we can coordinate.’ A Data.FI staff member said: ‘The region presented data to the Permanent Contingency Commission of Honduras, which transcends the situation room, and also coordinated actions with the departmental governor.’

Participants described how both the regional and national situation rooms boosted coordination with the national level, which contributed to improving data flow and quality. The appointment of national focal points to coordinate on specific topics also enhanced coordination between regional and national levels. In addition, participants reported how coordination with the national immunization program improved after the situation room intervention by harmonizing data sources and coverage reporting.

Participants in both regions highlighted support for the intervention by decision-makers and their consistent participation as a lynchpin of the situation room methodology. One participant said, ‘Together, the presence of decision-makers and the weekly progress reviews created, let’s say, a positive conditioning, meaning that staff felt committed to comply.’ Although the engagement of the regional director was key, the technical leadership of the surveillance unit was highlighted by the group as anchoring the intervention, since the leadership advocated for the activity, explaining its value from the onset. As one interviewee explained: ‘At the beginning, the director of the regional Health Surveillance Unit explained to us how this worked, and then, gradually, we became empowered.’

Most respondents indicated that situation rooms offered a space for direct communication and coordination of decision-makers across levels. In San Pedro Sula, the Health Network Coordinating Teams (ECORES)—focal points for all health networks coordinating with health facilities—routinely participated in regional situation room discussions. A Data.FI staff member noted: ‘Participating in the situation room is the only way ECORES are going to be able to respond in their localities. They wait for the situation room meetings, as they represent opportunities to learn. The representatives of ECORES are new staff but now they can perform general analyses and replicate them with the local staff.’ In the Central District, the ECORES structure does not exist; therefore, situation room participants communicated directly with health facilities using WhatsApp.

#### Governance of health data

This study relied on the definition of governance used by the Honduran MOH (see Table 2). The national concept of governance responds to how health system structures and instances are interrelated [19]. One of the regional authorities shared her frustrations about the early days of the pandemic when there was a lack of guidance from the national level on the procedures to be followed: ‘I imagine that up there [at the national level] they were in the same situation as us, not knowing what to do with the large number of cases, but well, we sorted it out along the way, but the [governance] void at the national level was felt at the time.’

Several participants in both regions mentioned that the regions were able to reduce the parallel efforts that had sprung up out of necessity, which resulted in discrepancies and duplication of efforts. For instance, one Data.FI interviewee explained that the region used to have an Excel-based tool parallel to the official health surveillance system (SVS). After situation room discussions, regional authorities issued guidance indicating the use of SVS as the main system for COVID-19 surveillance and the elimination of alternative tools. One interviewee noted that SVS was used in the Central District as the reporting system during the mpox outbreak.

Interviewees also described how situation room discussions helped remind them of existing systems. Through situation rooms, participants were able to adopt existing systems and use them in better ways to pull information from different systems for integrated analysis: ‘We have the health surveillance system, we have the vaccination system, we have the different systems that we have been using for the analysis of the different pillars of the situation rooms, we even have the HIV system now, so regarding information systems, we could say we have managed to triangulate information to unify the criteria.’ One participant illustrated how situation rooms supported staff to improve their management of vaccination systems: ‘Data.FI supported us to understand the SIVAC and be able to better manage the data and reach an agreement with the central level.’ Data management support may have had an impact on promoting health system governance: ‘We discovered that it is the way in which the technicians use the systems that the data varies sometimes because they modify some aspects or don't know how to use systems. With the help of Data.FI, technicians have been improving in this area, data management.’

Further, situation rooms constitute a forum to identify digital health needs. One interviewee mentioned that the region identified the need for an inventory management tool to track COVID-19 test stocks: ‘… when the region ran out of COVID-19 tests, a tool was developed and now it has been adopted for HIV.’

### What is the capacity of situation room staff to replicate the new situation room methodology to respond to other public health events or future threats?

In regard to the last study question, replicability of the new situation room health data use methodology was a main theme coming out of the interview analysis. Replicability is also evidence of successful capacity development. MOH staff indicated confidence and readiness to use the new methodology to solve other health issues: ‘Situation rooms can be replicated, not only during pandemics but for every process that we want to improve. It helps us organize, plan, identify strategies and act.’ This statement illustrates encouraging evidence that this intervention can support other health system areas beyond COVID-19, contributing more durably to the eHealth building blocks.

Both situation rooms provided examples of participants following the same methodology for their HIV work and adapting the methodology to review data in other areas, such as tuberculosis, dengue and maternal mortality. However, interviewees recognized that there might be challenges implementing the methodology in other regions due to the different levels of access to information and communication technology (ICT). For example, one person in San Pedro Sula mentioned that the methodology would be very useful to analyze progress made on leishmaniasis. However, this disease mostly occurs in mountainous rural areas where facilities lack information systems for reporting, preventing the analysis of data with the same frequency as other diseases. Another participant mentioned, ‘There will be other regions, especially those remote and with limited access to ICT to facilitate data management, where the intervention might not be fully implemented.’

Some participants described how the strengthened collaboration, planning, accountability and ability to create powerful visualizations could help health regions preserve the essence of the methodology and apply it to improve their response during health emergencies. One participant said, ‘We could set up situation room meetings because Data.FI taught us how to plan, how to assess progress and prioritize where to focus, how to prepare presentations and how to assign commitments to each one of us in a way that we could achieve milestones.’ Another provided an example of better preparedness as seen with mpox: ‘We used SVS for case identification, establishing epidemiological areas and deploying a more rapid and organized response. The organization of the response, as we did it during the situation room, helped because situation room participants knew what to do and how to do it.’

## DISCUSSION

Results from this study indicate that implementing subnational situation rooms in Honduras during the COVID-19 pandemic shows that these data review and coordination structures contributed to changing MOH staff attitudes and practices favoring more efficient and effective organization. Findings of this study align with elements of the COVID-19 Vaccine Digital Health Collaborative Learning Agenda’s theory of change. We noted evidence of the immediate outcomes in terms of data quality, analysis and use and indications of cross-sectoral outcomes of improved workforce capacity of regional staff and strengthened leadership and governance in both health regions, pointing to long-term positive effects for the health system. For instance, the qualitative results indicate that MOH staff were able to adopt and implement a new methodology, putting new data review processes into practice and leveraging the processes for current and future health responses. After the intervention, MOH staff place more value on and demand high-quality data for decision-making, enabling further digital health investments in systems development, interoperability and tools, such as dashboards. Altogether, we believe that a strengthened workforce, the most critical resource of the Honduran health system, and an enhanced regional leadership and governance will enable the country’s implementation of the digital health agenda.

### Workforce capacity

The evidence of self-reported performance improvement in the areas of data quality, analysis and use indicate that the capacity-blended coaching implemented by the intervention were successful. While the study lacks a proper baseline, the fact that half of questionnaire respondents attribute their proficiency and confidence in data use skills and knowledge to Data.FI indicates a possible positive impact. While staff turnover is high in Honduras, it is expected that MOH staff newly onboarded to the situation room approach will continue working inside the MOH for a significant period, potentially bringing their skills and experience to other MOH units and departments.

Participants reported less training, observation and practice in QI competencies. However, some interviews suggest that participants might have internalized and applied some QI methods without associating it to the theoretical concepts. Both quantitative and qualitative findings are encouraging given the situation room intervention's introduction of QI methods as a relatively new topic for most of the participants.

Peer collaboration was a fundamental element of the situation room intervention. Despite situation room participants having different education levels and professional experience, according to the qualitative results, they were able to discuss and question their ideas in a constructive way. By having real integration across units and departments, they were able to leverage information from different digital tools and systems, triangulate data and use quality data for guiding policy and programmatic decisions. Peer collaboration also promoted the development of soft skills. Teamwork facilitated decision-making through consensus as shared responsibilities removed decision-making from individuals or specific positions. In the longer term, this collaboration is expected to have cascade effects favoring the eHealth building blocks of workforce, leadership and governance, and even legislation, policy and compliance.

### Leadership and governance

We documented improvements in organizational capacity mostly in the areas of data analysis and visualization and peer collaboration. Improvements in leadership and governance were illustrated in the ways the two regional situation rooms fulfilled their mandates and fostered increased coordination and alignment of resources and processes with the national and local levels of the MOH and with external actors of the health system.

In terms of leadership, the qualitative results showed that the new methodology accelerates the decision-making process, easily turning situation room recommendations into action items through the presence of regional directors. Enhanced coordination with the national level was particularly important for the national immunization program, which was traditionally siloed and had limited synergy with other areas on the implementation of vaccination strategies. In addition, regional authorities were supportive of collaborating with the national level to ensure their teams report accurate information and are aligned with national guidelines and strategies.

In terms of governance, some interview participants and Data.FI staff suggested that the situation rooms were spaces to reinforce the use and understanding of existing information systems as well as to channel new systems or discuss digital health needs. Governance of health data constitutes a more abstract and longer-term aspect of the digital health environment in Honduras that needs further research, given the limited lack of use and understanding of digital health systems in the country. More structural interventions may be needed to overcome some of the obstacles that prevent a greater use of health systems for data collection and analysis (e.g. the lack of skilled human resources, connectivity, electricity, interconnected systems and legal framework).

### Sustainability and long-lasting contributions to the enabling environment

The fact that situation rooms are being used by both health regions to leverage data on other diseases and are addressing other health areas is a good indicator of a longer-term shift in the enabling environment. The true litmus test for the intervention would be for the two regions where situation rooms were implemented to demonstrate increased resilience and pandemic preparedness ultimately leading to better health outcomes. Although we may not have reached that point yet, the fact that three-quarters of respondents declared feeling very confident with participating in the creation of a situation room to respond to a new pandemic or health threat points in the right direction.

To ensure long-term sustainability of this digital health intervention, the new methodology and the corresponding National Data Use Strategy needs to be institutionalized through MOH national guidelines. Also, regions need to work hand-in-hand with the Directorate of Human Resources to design and roll out a training of trainers program that allows for regular training of existing and new staff. This type of institutionalized training could yield significant results in countries with high turnover rates, such as Honduras. If national rollout is considered, the government needs to be aware that lower levels of digital health enabling environment maturity could restrict the reach of this intervention to other health regions in the country.

### Limitations

The limitations of this study consist primarily of its limited size and scale. In addition, the data collection tool might have introduced bias in the form of recall and self-reporting. Also, respondents might have underestimated or overestimated their individual proficiency level. This limitation is critical given that the lack of a baseline restricts the attribution of the quantitative results to the situation room intervention.

Selection and desirability bias are other limitations of the study. The regional MOH offices have experienced high staff turnover, especially in the Central District health region. This suggests that not all interviewees received the same level of training or exposure to the intervention and, therefore, the impact on data use capacity may differ across individuals. Given these constraints, interview sampling was initially done randomly and complemented by convenience sampling. In addition, Data.FI's role as intervention implementer and evaluator during this study may have led to desirability bias.

This intervention started during a period of political instability, where the official activity launch and initial training took place just after a new government assumed power after 12 years in the opposition. In addition to the typical staff changes that occur during the first year of a new government, the implementation of situation rooms in Honduras was also affected by the seizure of regional facilities by organized groups in the Central District during the period of March to May 2023 and a strike of health care workers in San Pedro Sula in May 2023. These events impacted the intervention and this study's data collection process.

## CONCLUSIONS

This study points to successful implementation of situation rooms in Honduras with clear indications of replicability for other diseases and potential use for new health emergencies. In addition to using data for epidemiological surveillance, as was previously done, the region now uses data analysis and visualization for monitoring action plan progress, accountability and overall performance. This experience could help other ministries of health and donors promote situation rooms in countries that are still facing low vaccination coverage rates and where data quality prevents them from using information for decision-making. Moreover, the study results provide governments and international organizations with insights into the potential use of the situation room approach to improve evidence-based decision-making, coordination, and resource efficiency to reduce transmission and case-fatality rates when responding to future epidemics or pandemics.

## Data Availability

The data underlying this article cannot be shared publicly due to the privacy of individuals that participated in the study. The data will be shared upon reasonable request to the corresponding author and authorization by the Ministry of Health in Honduras.
